# Methylene Blue Reduces Electroretinogram Distortion and Ganglion Cell Death in a Rat Model of Glaucoma

**DOI:** 10.3390/biomedicines12091983

**Published:** 2024-09-02

**Authors:** Ronan Nakamura, Nicolás S. Ciranna, Juan C. Fernández, Rafael Peláez, Álvaro Pérez-Sala, Miriam Bobadilla, Juan J. López-Costa, César F. Loidl, Alfredo Martínez, Manuel Rey-Funes

**Affiliations:** 1 Institute of Cell Biology and Neurosciences “Prof. E. De Robertis”, Faculty of Medicine, University of Buenos Aires, Buenos Aires C1121ABG, Argentina; ronannak7@gmail.com (R.N.); nsc26@hotmail.com (N.S.C.); docfer2015@gmail.com (J.C.F.); jjlopez@fmed.uba.ar (J.J.L.-C.); cfloidl@yahoo.com.ar (C.F.L.); mreyfunes@fmed.uba.ar (M.R.-F.); 2 Biomarkers and Molecular Signaling Group, Neurodegenerative Diseases Area, Center for Biomedical Research of La Rioja (CIBIR), 26006 Logroño, Spain; rpelaez@riojasalud.es (R.P.); aperez@riojasalud.es (Á.P.-S.); mbobadilla@riojasalud.es (M.B.); 3 Angiogenesis Group, Oncology Area, Center for Biomedical Research of La Rioja (CIBIR), 26006 Logroño, Spain

**Keywords:** glaucoma, episcleral vein cauterization, methylene blue, rat model, scotopic electroretinography, pattern electroretinography, morphological analysis, inner retina thickness

## Abstract

Glaucoma is the second leading cause of blindness worldwide and is, in most cases, a consequence of elevated intraocular pressure (IOP), ultimately resulting in the death of retinal ganglion cells (RGCs). Current treatments are mostly focused on normalizing IOP, but we propose the additional use of neuroprotective agents, including methylene blue (MB), to block the loss of RGCs. Wistar rats were subjected to episcleral vein cauterization (EVC) in the left eye while the right eye was sham-operated. One week later, they were divided into two groups, which were injected with either 2.0 mg/kg MB or phosphate-buffered saline (PBS), twice a day, for 7 days. Fifteen days after surgery, rats were tested with scotopic electroretinography (ERG) or pattern electroretinography (PERG). After sacrifice, the number of RGCs and the thickness of the inner retina (IR) were evaluated both in the peripheral and central areas of the retina. Scotopic ERG showed a marked reduction (*p* < 0.0001) on the a- and b-wave amplitude and oscillatory potential (OP) complexity of the eyes subjected to EVC. These parameters were significantly (*p* < 0.01) restored by the application of MB. PERG indicated that EVC was responsible for a very significant decrease in N2 amplitude (*p* < 0.0001) and prolongation of N2 implicit time (*p* < 0.0001). Treatment with MB significantly restored N2 amplitude (*p* < 0.0001). In parallel with the ERG results, morphological analysis showed a significant loss of RGCs (*p* < 0.0001) and IR thickness (*p* < 0.0001) in both the peripheral and central retinas subjected to EVC, which was significantly prevented (*p* < 0.0001) by MB treatment. We have shown that MB treatment can be effective in preventing physiological and morphological hallmarks of optic neuropathy in a model of ocular hypertension, which faithfully recapitulates human open-angle glaucoma. Due to its high safety profile, this drug could therefore represent a new pharmacologic strategy to prevent vision loss in glaucoma patients.

## 1. Introduction

Optic neuropathy-related ocular lesions are responsible for major lifelong disabilities worldwide. This visual impairment implies significant morbidity for patients and an increasing economic burden to the health system and society in general. Glaucoma is the second leading cause of blindness worldwide, following cataracts. In 2020, around 76 million people were affected by this pathology, and these numbers are expected to rise to 111 million by the year 2040 [1]. Therefore, developing new treatment strategies for preventing vision losses becomes crucial.

Glaucoma constitutes a heterogeneous group of degenerative retinopathies affecting the inner retina. The histopathologic hallmark of these pathologies is the loss of retinal ganglion cells (RGCs) and concomitant thinning of the optic nerve fibre layer and optic nerve cupping. Because of the disposition of axons at the emergence of the optic nerve, this translates into progressive and irreversible loss of peripheral vision and eventually, in late stages, loss of central visual field and visual acuity. Given the oligosymptomatic nature of this disease, glaucoma is usually diagnosed at late stages when RGC loss is already significant [2].

There are many types of glaucoma, which are mostly classified in function of the pathophysiological mechanism of production and intraocular pressure (IOP) values, the latter accounting for the main and most frequent cause of glaucomatous degeneration. The most frequent form of glaucoma is primary open-angle glaucoma (POAG) which is responsible for more than 80% of cases [3,4]. The remaining forms include primary closed-angle glaucoma (PCAG), normotensive glaucoma and other causes of secondary glaucoma [5].

There are multiple aetiologies of glaucoma, but the main hallmarks of glaucoma are IOP elevation, vascular dysfunction and neurodegeneration, among others. Even though multiple theories have been proposed, currently there is a significant lack of advances related to the neurodegenerative process and the vascular component of glaucoma development. Also, the connection between the pressure gradient installed in the anterior segment and the development of optic neuropathy in the posterior segment is still a matter of study. The existence of normotensive forms of glaucoma, as well as the progression of glaucomatous changes after IOP has been normalized, supports the need to develop new therapeutic strategies, beyond IOP control, aimed to prevent the neurodegenerative process in the retina, which is more than just a mere sign of ocular hypertension [2].

Many experimental animal models have been established to study glaucoma pathophysiology and treatment. One of the most used models, probably because it best recapitulates the most common human form of glaucoma (POAG), is the episcleral vein cauterization (EVC) model, which was first proposed in 1995 [6]. This murine model has allowed significant advances in glaucoma knowledge, has been reproduced by multiple research groups [7,8,9], and has also been applied to other mammalian species [10].

The current paradigm of glaucoma treatment focuses exclusively in normalizing elevated IOP values, either through pharmacological (beta blockers, carbon anhydrase inhibitors, prostaglandins analogues, etc.) or, in some cases, surgical (iridotomy, laser iridoplasty, laser trabeculoplasty, trabeculectomy, etc.) approaches [2]. Furthermore, in the case of neovascular glaucoma, the use of anti-VEGF agents is recommended [11]. In addition, a number of novel approaches for the medical management of glaucoma are being investigated. These include the use of Rho kinase inhibitors, vasodilators, prostaglandin analogues, biodegradable implants, and nanotechnology [12]. Paradoxically, there is no approved treatment available to prevent or diminish the neurodegenerative retinal process, which is the mechanism ultimately responsible for blindness.

The free radical nitric oxide (NO) has been recognized as an important intercellular messenger in the eye and in the pathogenesis of glaucoma [13] but the effects of NO are somewhat paradoxical, depending on its physiological source. On the one hand, low levels of NO, such as those produced by the endothelial isoform of nitric oxide synthase (eNOS), increase blood flow and, thus, are beneficial for eye physiology [14] and some novel treatments based on providing low levels of NO through NO-donors are being investigated [15]. On the other hand, the high levels of NO generated by the inducible (iNOS), or occasionally the neuronal (nNOS), isoforms evoke neuronal cell death and vision losses [16]. Animal models suggest that an increasing IOP results in higher levels of NO in the eye that may induce RGC death [17,18]. In humans, evidence of increased levels of the three isoforms of NOS as a consequence of glaucoma have been shown in the optic nerve head [19]. Furthermore, treatment of POAG patients and matched healthy volunteers with a NOS inhibitor showed evidence of higher NO levels in the glaucoma group [20]. In consequence, inhibition of excessive NO levels may constitute a new avenue for glaucoma treatment and protection of the RGCs [21,22]. Most physiological actions of NO are mediated by the formation of the second messenger cGMP, which is produced by the enzyme guanylyl cyclase (GC) [23]. In previous studies, our group has demonstrated that methylene blue (MB), a drug that is used in the clinic with a high safety profile [24], is a guanylyl cyclase inhibitor, free radical scavenger [25], and inhibitor of both constitutive and inducible isoforms of NOS [26,27], can protect the retina from RGC death [28,29]. The proposed mechanism of action for this effect was the inhibition of NOS/GC/cGMP by MB, followed by the reduction of NO levels, and blockade of nitrosative damage caused by nitrogen reactive species [16].

The main objective of this work was to develop an efficacious treatment to prevent glaucoma-related retinal lesions. For that purpose, the pharmacological effect and efficacy of MB was evaluated in a rat EVC model of ocular hypertension, analysing its influence through modifications in two hallmarks of optic neuropathy, e.g., electroretinogram and morphological changes in the retina.

## 2. Materials and Methods

### 2.1. Ocular Hypertension Glaucoma Model Using Episcleral Vein Cauterization

Young (8-week-old) male Wistar rats (n = 50) with genetic quality and sanitary certification from the animal facility of our institution were cared for in accordance with guidelines published in the *ARVO Statement for the Use of Animals in Ophthalmic and Vision Research*. All procedures were approved by the Ethical Committee of CICUAL (Comité Institucional para el Uso y Cuidado de Animales de Laboratorio, Facultad de Medicina, Universidad de Buenos Aires, Buenos Aires, Argentina. Resolution RESCD-2023-1408-E-UBA-DCT#FMED). Animals were kept under standard laboratory conditions, with light/dark cycles of 12/12 h, a constant temperature of 21.0 ± 2.0 °C, and food and water provided ad libitum.

**Figure 1:**
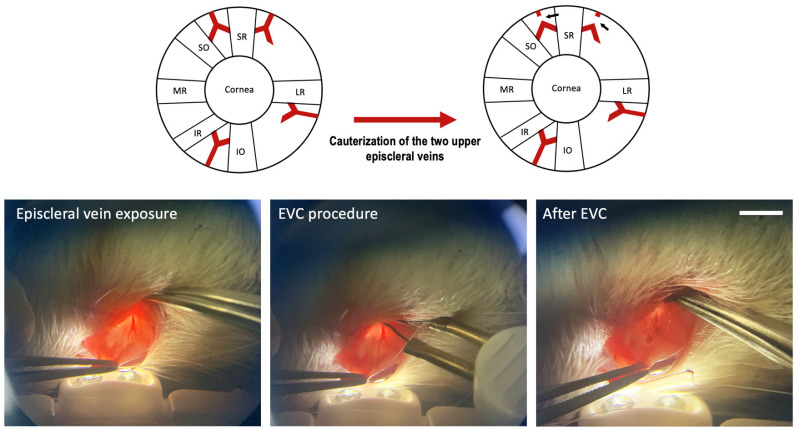
Schematic drawing of the localization of the episcleral veins on the eye and effect of the cauterization procedure (upper row). Small black arrows indicate the cauterized veins. SR: superior rectus muscle, SO: superior oblique muscle, MR: medial rectus muscle, IR: inferior rectus muscle, IO: inferior oblique muscle, LR: lateral rectus muscle. Actual photographs of the procedure are shown in the lower row. Size bar = 5 mm.

The EVC protocol was performed as described [6], with slight modifications. Briefly, rats were anesthetized with an intraperitoneal injection of 40 mg/kg ketamine (Ketamina 50, Holliday-Scott S.A., Buenos Aires, Argentina) and 5 mg/kg xylazine (Xilacina 20 Richmond, Laboratorios Richmond, Buenos Aires, Argentina). A small incision was performed on the superior bulbar conjunctiva, and then another on the Tenon capsule, of the left eye. Connective tissue was carefully dissected and episcleral veins were exposed (Figure 1). The two episcleral veins adjacent to the superior rectus muscle were identified by pupillary transillumination. They were immediately cauterized using a hand-held electrocautery (Bovie Medical Corp., Cleanwater, FL, USA), taking caution to not produce excessive thermic damage on the adjacent tissues. Surgical wound closure was performed by layers. The contralateral eye was sham-operated: the episcleral veins were exposed but not cauterized. After surgery, intraperitoneal Tramadol 5 mg/kg (John Martin S.R.L., Buenos Aires, Argentina) and topic ophthalmic eritromicin ointment (Eritromicina Elea, Elea, Buenos Aires, Argentina) were applied as analgesic and antibiotic prophylaxis, respectively. Animals were checked daily for signs of pain or discomfort.

All animals were subjected to EVC in the left eye, whereas the right eye was sham-operated, as described [6] (Figure 1).

**Figure 2:**
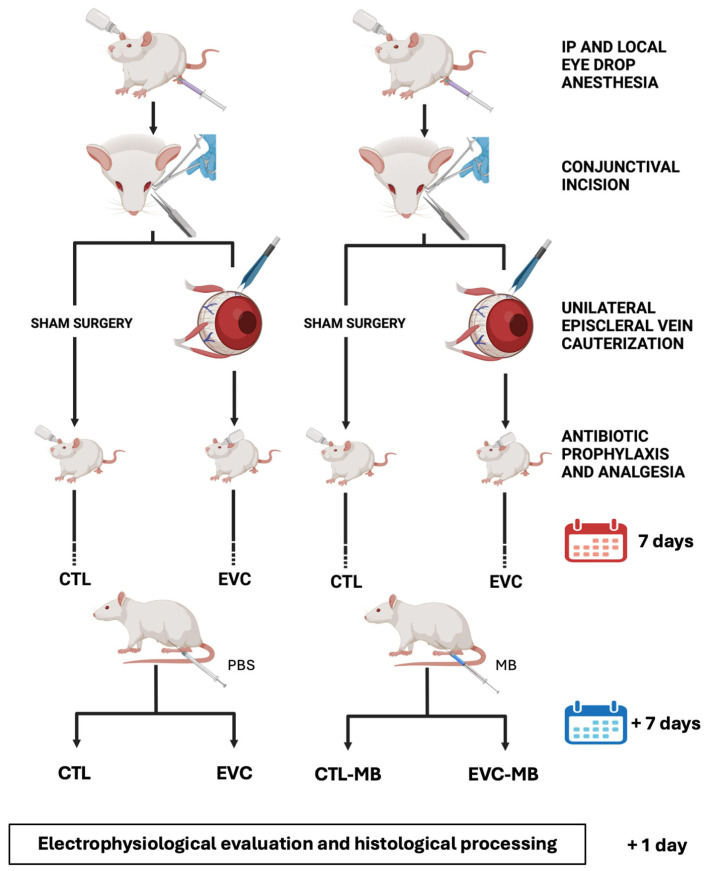
Schematic drawing of the experimental procedure. The left eyes were subjected to episcleral vein cauterization (EVC) while the right eyes were sham-operated. Seven days later, rats were injected, twice a day for 7 days, with either PBS or 2.0 mg/kg MB. One day after finishing the treatment, animals were subjected to ERG and sacrificed. Four experimental groups of eyes were generated: CTL, EVC, CTL-MB and EVC-MB.

Seven days post-surgery, animals were randomly distributed into two groups. Half of the rats started intraperitoneal treatment with 2.0 mg/kg MB (Sigma, St. Louis, MO, USA), twice a day for seven days, while the other half received the same volume of vehicle (PBS) (Figure 2). One day after finishing the treatments, all animals were subjected to electrophysiological evaluation with one of two different paradigms: scotopic electroretinography (ERG, n = 10 per group) or pattern electroretinography (PERG, n = 15 per group) (see below). Finally, all animals were sacrificed and tissues from the ERG group were collected for histological studies (see below).

### 2.2. Scotopic Electroretinography (ERG)

Fifteen days after surgery, the first group of rats (n = 10 per group) was subjected to electroretinography, as described in [30]. Briefly, after an overnight adaptation in the dark, rats were anaesthetized with ketamine/xylazine under dim red illumination. An ophthalmic solution containing 5% phenylephrine hydrochloride and 0.5% tropicamide (Fotorretin, Poen, Buenos Aires, Argentina) was used to dilate the pupils and a local proparacaine ointment (Poen-caina, Poen, Buenos Aires, Argentina) was applied over the cornea as a local anaesthetic. Rats were placed facing the stimulus at a distance of 25 cm in a highly reflective environment. Scotopic electroretinograms (ERGs) were recorded from both eyes simultaneously, and 20 responses were collected to flashes of unattenuated white light (1 m, 1 Hz) from a photic stimulator set at maximum brightness. The registered response was amplified (9 cd s/m^2^ without filter), filtered (1.5-Hz low-pass filter, 500 Hz high-pass filter, notch-activated), and averaged (Akonic BIO-PC, Buenos Aires, Argentina). The a-wave was estimated as the difference in amplitude between the recording at onset and the trough of the negative deflection, and the b-wave amplitude was calculated from the trough of the a-wave to the following peak. To calculate oscillatory potentials (OPs), the same photic stimulator was used with filters of high (300 Hz) and low (100 Hz) frequency. The amplitudes of the OPs were recorded by using the peak-to-trough method.

### 2.3. Pattern Electroretinography (PERG)

Fifteen days after surgery, another group of rats (n = 15 per group) was subjected to pattern electroretinography, as described in [31], with slight modifications. Briefly, rats were anaesthetized under dim light with an intraperitoneal injection of ketamine/xylazine, as above. A local proparacaine ointment (Poen-caina, Poen, Buenos Aires, Argentina) was applied over the cornea. Rats were placed at a 45° angle, so one of their eyes was facing the stimulus at a distance of 20 cm. Transient PERGs were recorded from each eye, separately. The visual stimulus was generated by commercial software (Akonic BIO-PC, Buenos Aires, Argentina) and displayed on a CRT monitor (SyncMaster 591s, Samsung, Suwon-si, South Korea). It consisted of a black and white full 10 × 8 checkboard with a 50% duty cycle that alternated at a temporal frequency of 2 reversals per second (1.00 Hz) and a spatial frequency of 0.068 cycles per degree. Contrast was maintained at 90%, and mean luminescence of the projected display was 50 cd/m^2^. Duration of the stimulus was 300 milliseconds and results were the average of 100 cycles. N2 wave amplitude was calculated as the difference between the P1 peak and the following through. N2 peak latency is the time from the initiation of the stimulus until the N2 through occurs.

### 2.4. Histology and Morphological Evaluation

After completing the electroretinography, the rats were deeply anaesthetized with an intraperitoneal injection of ketamine/xylazine and sacrificed by decapitation. Eyes were enucleated and fixed in 4.0% paraformaldehyde for 48 h at 4 °C, dehydrated, paraffin-embedded, and sectioned (3.0 μm-thick). Sections were stained with haematoxylin and eosin (H&E). Before assays, care was taken in selecting anatomically matching areas among animals for an accurate analysis. To avoid variations in the quantification process, all images were obtained the same day and under the same light and contrast conditions. The number of RGCs per 500 μm of retinal length and inner retinal (IR) thickness was calculated for each experimental group (6 eyes, 7 fields per eye, for a total of 42 fields per group), as previously reported [32]. The IR is described as the sum of the ganglion cell layer, the inner limiting layer, and the optic nerve fibre layer, and its thickness usually correlates with the severity of different retinopathies [33]. Two different regions were evaluated in each retina, peripheral and central. The distinction between both was performed based on whole-retinal thickness, as reported [34]. A value of 220 μm was selected as the threshold for dividing the central retina (>220 μm) from the peripheral retina (<220 μm). Quantification of these parameters was performed using ImageJ software, version 1.53 g (Wayne Rasband & Co., National Institute of Health, Bethesda, MD, USA) in two different areas, peripheral and central retina, in all eyes.

### 2.5. Statistical Analysis

All data were analysed with GraphPad Prism, version 10.2.3, software and were considered statistically significant when *p* < 0.05. Values are expressed as means ± SEM. All data sets were evaluated for normality (Shapiro–Wilk) and homoscedasticity (Levene). Normally distributed data were evaluated by one-way ANOVA followed by the Dunnet’s (Bonferroni) post hoc test, while data not following a normal distribution were analysed with the Kruskal–Wallis test followed by the Mann–Whitney U test. In this case, all data sets followed a normal distribution.

## 3. Results

### 3.1. Surgery and Treatment Groups

Seven days post-surgery, half of the rats started intraperitoneal treatment with 2.0 mg/kg MB, while the other half received the same volume of vehicle (PBS). This design generated four experimental eye groups (Figure 2): (i) sham-operated, injected with vehicle (CTL), (ii) EVC-operated, injected with vehicle (EVC), (iii) sham-operated, injected with MB (CTL-MB) and (iv) EVC-operated, injected with MB (EVC-MB).

### 3.2. Methylene Blue Restores Electroretinogram Patterns

One day after finishing the treatments, all animals were subjected to electrophysiological evaluation with one of two different paradigms: scotopic electroretinography (ERG, n = 10 per group) or pattern electroretinography (PERG, n = 15 per group).

#### 3.2.1. Scotopic Electroretinography

**Figure 3:**
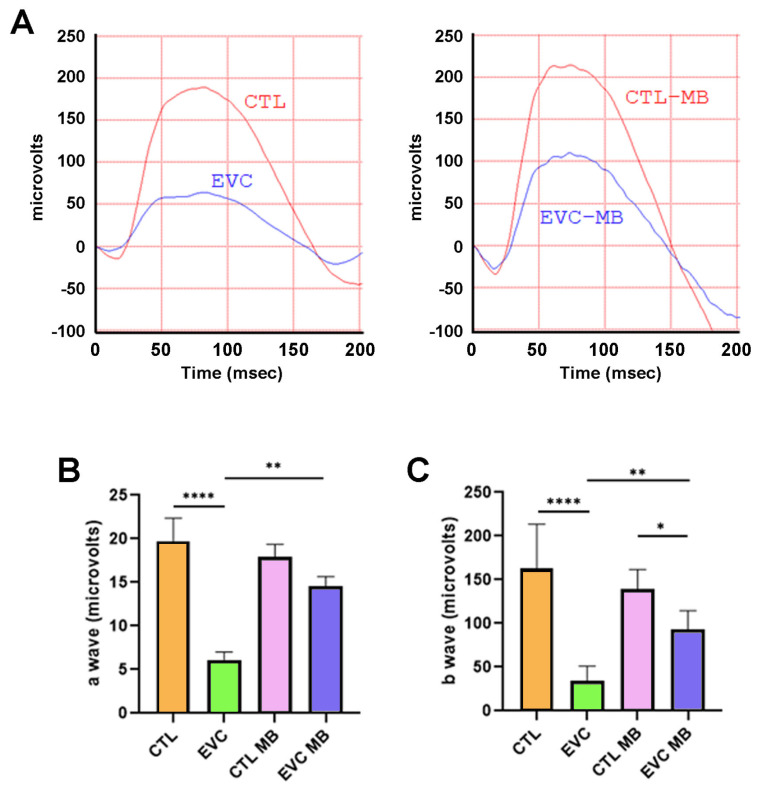
MB prevents changes in the scotopic ERG induced by EVC. (**A**) Representative electroretinograms taken 15 days post-surgery for animals that received either PBS or MB. The red line corresponds to the right eye, whereas the blue line is the recording of the left eye. (**B**) Amplitude of the a-wave in the four experimental groups. (**C**) Amplitude of the b-wave in the four experimental groups. In both cases, EVC induced a significant decrease in the a- and b-wave compared to control (CTL), whereas MB partially or completely prevented it. Each bar represents the mean ± SEM of 10 animals. One-way ANOVA. Asterisks indicate significant differences: * *p* < 0.05; ** *p* < 0.01; **** *p* < 0.0001.

Scotopic ERG showed that those eyes subjected to the EVC procedure had a significant (*p* < 0.0001) reduction of the a- and b-wave amplitude compared to control sham-operated eyes (CTL group) (Figure 3A–C). Treatment with MB of sham-operated eyes (CTL-MB) had no impact on these parameters (Figure 3A–C). On the other hand, treatment with MB of eyes subjected to EVC (EVC-MB) resulted in a significant preservation of the a-wave amplitude (*p* = 0.0059) and b-wave amplitude (*p* = 0.0010) versus the EVC group (Figure 3A–C).

#### 3.2.2. Oscillatory Potentials

**Figure 4:**
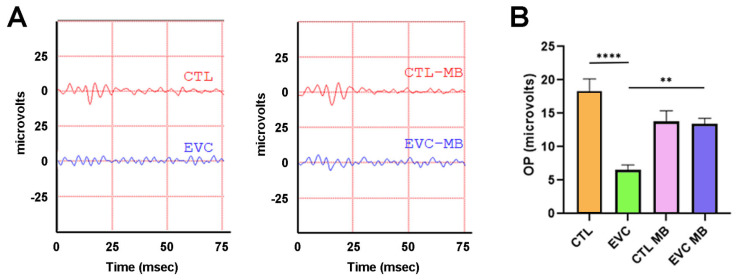
MB prevents changes in the oscillatory potentials induced by EVC. (**A**) Representative oscillatory potentials (OP) of the electroretinograms taken 15 days post-surgery for animals that received either PBS or MB. The red line corresponds to the right eye whereas the blue line is the recording of the left eye. (**B**) Sum of amplitudes of the OP in the four experimental groups. EVC induced a significant decrease in the OP compared to control (CTL), whereas MB treatment prevented it. Each bar represents the mean ± SEM of 10 animals. One-way ANOVA. Asterisks indicate significant differences: ** *p* < 0.01; **** *p* < 0.0001.

Similar observations were made when studying the OP recordings. Compared to controls (CTL), EVC surgery produced a significant (*p* < 0.0001) loss of complexity in the OP patterns whereas MB treatment significantly (*p* = 0.0046) preserved OP waveform complexity in a manner undistinguishable from controls. Similarly to the a- and b-waves, application of MB to sham-operated controls had no impact on the OPs (Figure 4A,B).

#### 3.2.3. Pattern Electroretinography

**Figure 5:**
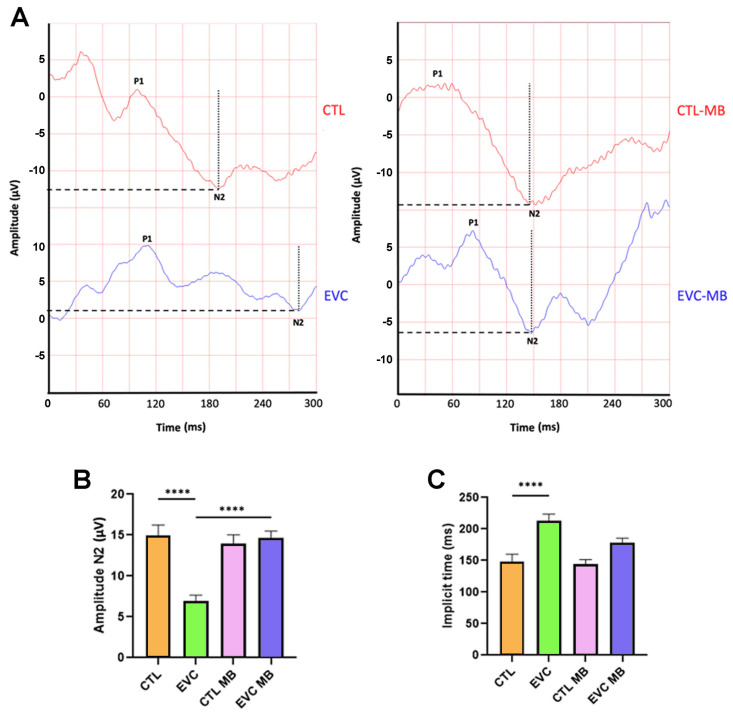
MB prevents changes in the PERG induced by EVC. (**A**) Representative pattern electroretinograms taken 15 days post-surgery for animals that received either PBS or MB. The red line corresponds to the right eye, whereas the blue line is the recording of the left eye. (**B**) Amplitude of the N2 wave in the four experimental groups. (**C**) N2 implicit time in the four experimental groups. EVC induced a significant decrease in the N2 amplitude compared to the control (CTL), whereas it increased the N2 implicit time. MB treatment completely restored N2 amplitude but only partially restored its implicit time. Dotted lines indicate the amplitude of the N2 wave. Dashed lines show the N2 implicit time. Each bar represents the mean ± SEM of 15 animals. One-way ANOVA. Asterisks indicate significant differences: **** *p* < 0.0001.

PERGs were also recorded to evaluate with higher sensitivity the RGCs’ function and integrity. Two main parameters were evaluated because of their relevance for glaucoma [35,36]: amplitude and implicit time of the N2 wave (Figure 5A). Eyes subjected to EVC surgery presented a significant (*p* < 0.0001) reduction in N2 amplitude in relation to the controls (CTLs). In contrast, treatment of eyes subjected to EVC with MB (EVC-MB) resulted in a significant (*p* < 0.0001) preservation of N2 amplitude, with values that were undistinguishable from the controls (CTL-MB). As with regular ERGs, MB had no effect on sham-operated eyes (Figure 5A,B). In the case of N2 implicit time, EVC caused a significant (*p* < 0.0001) delay in this parameter. The application of MB was not able to induce a significant recovery of N2 implicit time (Figure 5A,C).

### 3.3. Methylene Blue Prevents Loss of RGC and IR Thickness

In the histological slides stained with H&E, the number of RGCs and IR thickness in the peripheral and central retinal sections were analysed.

#### 3.3.1. Peripheral Retina Morphology

**Figure 6:**
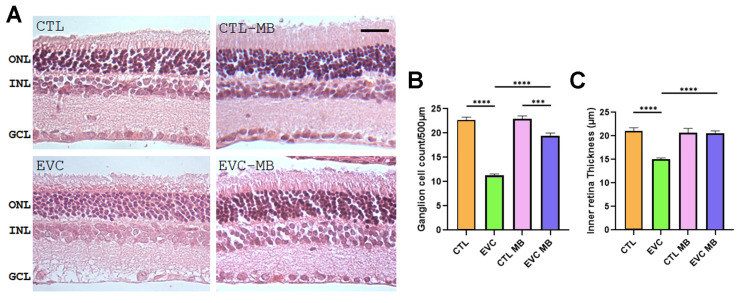
Death of RGCs and inner retina thickening are prevented by MB treatment in the peripheral retina. Representative histological images of the peripheral retina of animals of the 4 experimental groups, taken 15 days after surgery, and stained with hematoxylin–eosin (**A**). Three layers of the retina are labelled in the pictures for reference: outer nuclear layer (ONL), inner nuclear layer (INL), and ganglion cell layer (GCL). Scale bar = 50 μm. Quantification of the number of RGCs (**B**) and IR thickness (**C**) are shown as histograms. Bars represent the mean ± SEM of all samples (6 eyes per group). One-way ANOVA followed by Bonferroni post hoc test. Asterisks represent statistically significant differences: *** *p* < 0.001; **** *p* < 0.0001.

At the peripheral retina, EVC-operated eyes had a significant (*p* < 0.0001) loss of RCGs compared with the CTL group (Figure 6A,B). In contrast, MB treatment of EVC-operated eyes showed a significant (*p* < 0.0001) preservation of RGC count, although total values were still lower than in the controls (CTL-MB) (*p* < 0.001). MB treatment did not modify the number of RGCs in sham-operated eyes (CTL-MB) (Figure 6A,B).

Regarding IR thickness, there was also a significant (*p* < 0.0001) thinning of this layer generated by EVC surgery (Figure 6A,C). MB treatment (EVC-MB group) completely prevented (*p* < 0.0001) this modification and was indistinguishable from the controls (CTL-MB). As with other parameters, MB had no effect on sham-operated eyes (Figure 6A,C).

#### 3.3.2. Central Retina Morphology

**Figure 7:**
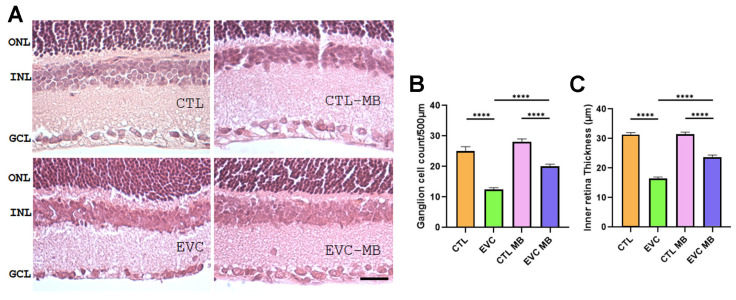
Death of RGCs and inner retina thickening are prevented by MB treatment in the central retina. Representative histological images of the central retina of animals of the 4 experimental groups, taken 15 days after surgery, and stained with hematoxylin–eosin (**A**). Three layers of the retina are labelled in the pictures for reference: outer nuclear layer (ONL), inner nuclear layer (INL), and ganglion cell layer (GCL). Scale bar = 50 μm. Quantification of the number of RGCs (**B**) and IR thickness (**C**) are shown as histograms. Bars represent the mean ± SEM of all samples (6 eyes per group). One-way ANOVA followed by Bonferroni post hoc test. Asterisks represent statistically significant differences: **** *p* < 0.0001.

The morphological changes observed in the central retina were similar to those described in the peripheral retina, but with some differences. Here, there was also a clear reduction (*p* < 0.0001) in both RGC counts and IR thickness due to EVC surgery (Figure 7A–C). Both parameters were significantly (*p* ≤ 0.0001) preserved by treatment with MB but, in this case, both parameters were lower than the controls (CTL-MB) (*p* < 0.0001). As in the peripheral retina, the application of MB to sham-operated eyes had no effects (Figure 7A–C).

## 4. Discussion

In this study, we have demonstrated that MB treatment can be effective in preventing physiological and morphological hallmarks of optic neuropathy in a rat model of ocular hypertension, which faithfully recapitulates human POAG, the most common clinical presentation of glaucoma [3]. This study follows our previous work, where we showed that MB treatment had a beneficial impact in the retinal integrity of newborns subjected to perinatal asphyxia [28,29]. Based on these previous findings, we decided to apply this novel neuroprotective strategy to a model of hypertensive glaucomatous neuropathy induced by EVC, generating data that may change the current paradigm of glaucoma treatment.

Interestingly, MB exhibits a high safety profile [24,37] and is approved for clinical use as an antidote of poison-induced methemoglobinemia [38], in norepinephrine refractory hypotension [39], and for the surgical management of hyperparathyroidism [37], among other applications. MB is on the World Health Organization’s *List of Essential Medicines*, which gives the most effective and safe medicines needed in a health system [40]. The only known incompatibility with MB is the use of serotoninergic drugs [41], so special care should be taken with glaucoma patients who are treated with antidepressants.

Scotopic ERG findings showed that EVC resulted in a very significant reduction in the amplitude of both the a- and b-waves, which represent the electrical recordings of the photoreceptors’ and the bipolar/Müller glia cells’ activity, respectively [42]. Even though there is no direct damage to bipolar cells and Müller glia in glaucoma, their electrical activity is most likely affected by RGC compromise, therefore resulting in b-wave amplitude reduction. The implication of the RGCs is the expected electrophysiological hallmark of glaucoma, given the compression of RGCs’ axons at the *lamina cribosa* due to ocular hypertension (OHT). This compression produces cellular stress by axoplasmic flux blockade, decrease in transport of neurotrophic factors to the soma and, eventually, neuronal cell death [2,43,44]. The administration of MB resulted in a significant restoration of the ERG’s waveform and amplitude for both waves. These findings account for inner retina neuroprotection, especially of the RGCs, which are the cells most affected by this pathology. This is also in agreement with the morphological results, which showed a significant preservation of the number of RGCs in the retinas treated with MB. As for the a-wave findings, no morphological alterations were observed at the photoreceptor layer, as was expected of an OHT model. Therefore, the electrophysiological changes in the a-wave are most likely a result of retrograde electrical current influences from the RGCs that do not result in photoreceptor structural damage, in a similar manner to that described for the b-wave and bipolar cells. This phenomenon has already been described in other models of retinopathy [29,45,46].

In addition, scotopic OPs were also significantly altered by EVC. Scotopic OPs are a high-frequency low-voltage component of the ERG, which correlates tightly with the b-wave, and may be seen as slight indentations in its ascending limb. However, their origin is slightly different from the cells that produce the b-wave, and depends on the electrical activity of bipolar and amacrine cells, instead [47,48]. It is well known that OPs constitute a very sensitive parameter of vascular dysfunction, including that produced by hypoxia, in the retina, and they even precede a- and b-wave alterations [47,49,50]. As for the exact significance of OP modifications in glaucoma, the verdict is still inconclusive, though these alterations have been previously described [51,52,53]. In our experiment, we found that the OPs in the EVC group had a significantly lower complexity in relation to the controls, something that usually indicates the presence of some retinopathy/neuropathy [32,54]. The distortion of the OP pattern found in glaucomatous eyes could be a consequence of the initial damage to the RGCs, which would result in a synaptic dysfunction at the inner plexiform layer, where RGCs connect with bipolar and amacrine cells. Furthermore, due to hemodynamic alterations produced by the IOP, areas of ischemia/hypoxia may occur in the inner retina, thus generating OP distortion. Interestingly, this distortion was completely prevented by the treatment with MB, suggesting a clear neuroprotective effect for this drug.

To further characterize the effects of our glaucoma model and the posterior MB effects in the RGCs, we performed PERG analyses, which have been described as the most sensitive method to evaluate RGC function [31], even before significant losses of RGCs and vision field are evident [35,36]. N2 wave (equivalent to human PERG N95 wave) amplitude and its implicit time were quantified. This wave is originated exclusively from RGCs [55] and, chronologically, is the first electrophysiological alteration found in glaucoma pathophysiology. As expected, EVC eyes showed a significant reduction in N2 amplitude compared to controls, whereas N2 implicit time was significantly prolonged. Increased N2 peak latency has been associated with RCG dysfunction, even before cell death occurs [56]. On the other hand, MB treatment showed a significant preservation of N2 amplitude in relation to EVC, with values identical to controls, thus confirming the great potential of this drug as a neuroprotective agent. Regarding N2 implicit time, MB did not produce a significant recovery. Nevertheless, the N2 implicit time of MB-treated EVC eyes was also indistinguishable from MB-treated controls. These findings suggest that MB significantly prevented RGC death, but some of these viable cells may still have signs (slightly increased N2 implicit time) of slow conductance and cell dysfunction, probably due to OHT-mediated cellular stress.

Histological evaluation of H&E retinal sections showed a significant loss of RGC counts and IR thickness in the EVC group in comparison to controls. These morphological hallmarks coincided with the electrophysiological findings, for both ERG and PERG. Treatment with MB translated into a highly significant restoration of most of these parameters supporting the relevance of adding this drug to the current treatments for glaucoma.

This study has some limitations. First, this work was designed as a proof-of-concept, so experiments were carried out exclusively in male rats to reduce potential variability due to sex and hormonal influences. In humans, men have a higher burden of glaucoma than women [57] and there is growing evidence that sex hormones play a role in the pathophysiology of glaucoma [58]. Future studies will show whether these hormones have any influence in the efficacy of the MB treatment. In addition, MB is a very soluble drug. This characteristic has advantages and disadvantages. The main advantage is shown in our study, where, by using an intraperitoneal injection, we were able to influence retinal pathophysiology, demonstrating that MB can easily cross the blood–ocular barrier. But this behaviour also poses a clear pharmacokinetic disadvantage, namely that MB will remain in the eyeball only for short periods of time. Therefore, if we want to maintain a chronic treatment with MB, very frequent injections (twice a day in our experiment) will be necessary. Since glaucoma is a chronic disease that often requires lifelong management, we are actively pursuing new strategies to increase MB´s half-life in the eye and investigating alternative administration routes, including topic application.

## 5. Conclusions

In summary, we have shown MB to be an effective treatment for reducing electrophysiological and morphological alterations generated by ocular hypertension and glaucomatous retinal damage. Due to its high safety profile, this drug could therefore represent a new pharmacologic strategy to prevent vision losses in glaucoma patients. Nevertheless, further research is needed to solve the current pharmacokinetic obstacles that this molecule represents for the treatment of a chronic condition such as glaucoma.

## Data Availability

The raw data supporting the conclusion of this article will be made available by the authors, without undue reservation.
